# Development and evaluation of a deep learning segmentation model for assessing non-surgical endodontic treatment outcomes on periapical radiographs: A retrospective study

**DOI:** 10.1371/journal.pone.0310925

**Published:** 2024-12-31

**Authors:** Dennis Dennis, Siriwan Suebnukarn, Sothana Vicharueang, Wasit Limprasert

**Affiliations:** 1 Faculty of Dentistry, Universitas Sumatera Utara, Medan, Indonesia; 2 Faculty of Dentistry, Thammasat University, Pathum Thani, Thailand; 3 StoreMesh, Thailand Science Park, Pathum Thani, Thailand; 4 College of Interdisciplinary Studies, Thammasat University, Pathum Thani, Thailand; New York University College of Dentistry, UNITED STATES OF AMERICA

## Abstract

This study aimed to evaluate the performance of a deep learning-based segmentation model for predicting outcomes of non-surgical endodontic treatment. Preoperative and 3-year postoperative periapical radiographic images of each tooth from routine root canal treatments performed by endodontists from 2015 to 2021 were obtained retrospectively from Thammasat University hospital. Preoperative radiographic images of 1200 teeth with 3-year follow-up results (440 healed, 400 healing, and 360 disease) were collected. Mask Region-based Convolutional Neural Network (Mask R-CNN) was used to pixel-wise segment the root from other structures in the image and trained to predict class label into healed, healing and disease. Three endodontists annotated 1080 images used for model training, validation, and testing. The performance of the model was evaluated on a test set and also by comparison with the performance of clinicians (general practitioners and endodontists) with and without the help of the model on independent 120 images. The performance of the Mask R-CNN prediction model was high with the mean average precision (mAP) of 0.88 (95% CI 0.83–0.93) and area under the precision-recall curve of 0.91 (95% CI 0.88–0.94), 0.83 (95% CI 0.81–0.85), 0.91 (95% CI 0.90–0.92) on healed, healing and disease, respectively. The prediction metrics of general practitioners and endodontists significantly improved with the help of Mask R-CNN outperforming clinicians alone with mAP increasing from 0.75 (95% CI 0.72–0.78) to 0.84 (95% CI 0.81–0.87) and 0.88 (95% CI 0.85–0.91) to 0.92 (95% CI 0.89–0.95), respectively. In conclusion, deep learning-based segmentation model had the potential to predict non-surgical endodontic treatment outcomes from periapical radiographic images and were expected to aid in endodontic treatment.

## Introduction

Modern endodontic treatments are highly effective in saving teeth that might otherwise need to be extracted. However, like any medical procedure, there is always a chance of failure. Non-surgical endodontic treatment, commonly referred to as root canal treatment, is a dental procedure aimed at treating infection or damage within the tooth’s pulp without the need for surgical intervention. The outcome of endodontic treatment is crucial especially if the clinical decision regarding a compromised tooth is to be made either through root canal treatment or extraction [1]. Root canal treatment outcomes are dominantly influenced by the nature of prior dynamic host/infection interaction (pre-operative patient factors), the active efficacy of the operators’ root canal treatment protocol to sustain a microbial ecological shift and resolve periapical inflammation (intra-operative treatment factors), and the passive ability of the functional tooth and its restoration margin to maintain its integrity to resist infection reversal (postoperative restorative factors) [2–4].

Evaluating the treatment outcomes of non-surgical endodontic treatment involves several methods, focusing primarily on clinical assessments and imaging techniques [1]. The most commonly used imaging modalities are parallel digital periapical radiographs and cone beam computed tomography (CBCT). The combination of clinical assessments, parallel digital periapical radiographs, and CBCT provides a comprehensive approach to evaluating the outcomes of non-surgical endodontic treatment. Traditional two-dimensional radiographs remain a staple due to their wide availability in dental practices, low radiation dose, and provide rapid feedback for immediate diagnosis and treatment planning. CBCT provides three-dimensional imaging of teeth, bones and surrounding structures, offering invaluable information in complex cases [2].

One of the main factors that may influence outcomes of endodontic treatment is the effect of tooth integrity [5]. Preoperative clinical evidence of compromised tooth structure, such as in the form of reduced amount, distribution, quality (sclerosed dentine) or integrity (cracks) of enamel or dentine may reduce the prospect of periapical healing [1, 5, 6]. Often, endodontically treated teeth experience tissue loss due to prior pathology and compromise the mechanical integrity of the remaining tooth structure [6]. This important factor is considered further under postoperative factors. Fractures of restored endodontically treated teeth are a common occurrence in clinical practice. Severely fractured teeth that cannot be salvaged are typically extracted and replaced with implants, bridges, or dentures to restore function and aesthetics. Hence, predicting potential failure during the preoperative phase of non-surgical endodontic treatment is crucial to ensure that patients receive the most appropriate treatment.

Artificial intelligence (AI) technology, especially deep learning, has demonstrated significant potential in the field of medical and dental imaging analysis, including applications in oral health care [7, 8]. As AI technology continues to advance, its integration into dental practice can contribute to more accurate diagnoses, enhanced treatment planning, and ultimately improved patient outcomes [9]. AI can contribute to the improvement of diagnosis and treatment that can lead to an increase in the success of endodontic treatment outcomes [10]. Deep learning, or deep neural networks, is built with multiple layers of convolutional neural networks designed to autonomously learn and extract features from image data. Deep learning models can outperform or match the diagnostic accuracy of dental specialists in identifying and diagnosing endodontic issues, such as root canal abnormalities [11] and periapical lesions [12–14]. The integration of deep learning into endodontic treatment is a promising trend that has the potential to revolutionize the field by improving diagnostic accuracy and enhancing treatment planning.

This aim of this study was to develop and evaluate non-surgical endodontic treatment outcome prediction model using deep learning technology. A Mask R-CNN segmentation algorithm was implemented to outline and separate the root from other structures on preoperative periapical radiographic images with known treatment results and to predict class label into healed, healing and disease. The performance of the Mask R-CNN model was evaluated on a test set and also by comparison with the performance of clinicians (general practitioners and endodontists) with and without the help of the model on independent periapical radiographs. The model evaluation was based on precision, recall, F1 score, the area under the precision-recall curve (AUC), and mean average precision (mAP). The clinician evaluation was based on sensitivity, specificity, precision, and mAP. The hypothesis posited that the integration of the Mask R-CNN model with clinicians would improve the accuracy of predicting endodontic treatment outcomes on preoperative periapical radiographs compared to predictions made by clinicians alone. The proposed model is expected to provide AI second opinions for preoperative endodontic treatment planning to ensure that patients receive the most appropriate treatment.

## Materials and methods

**Fig 1:**
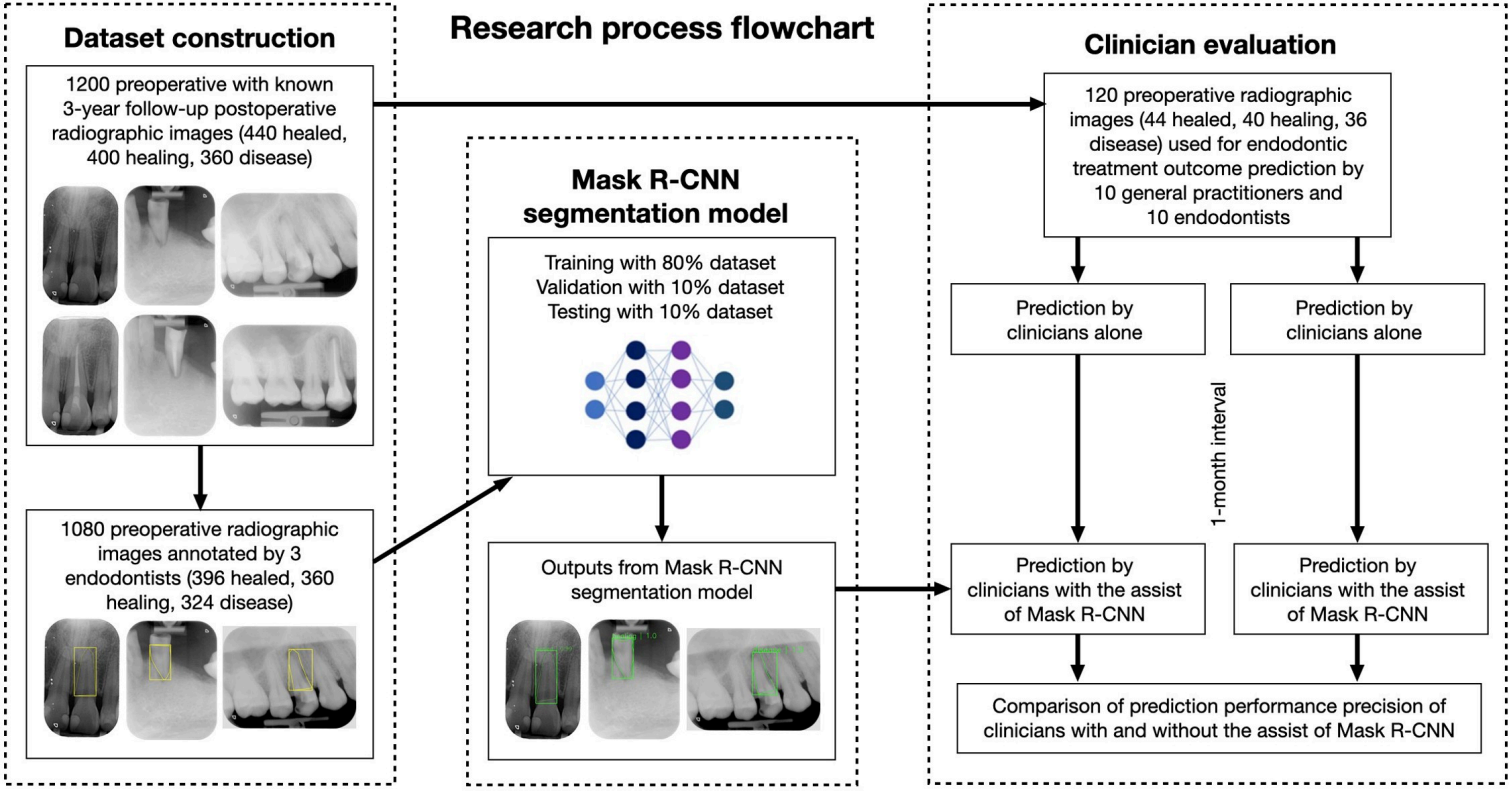
The flowchart of dataset construction, Mask R-CNN model building and clinician evaluation.

This study employed a retrospective experimental design to develop and evaluate a deep learning model for assessing non-surgical endodontic treatment outcomes on periapical radiographs. The study involves two key phases: model development (retrospective phase) and model evaluation (experimental phase) (Fig 1). This study was approved by the Human Research Ethics Committee of the author’s University (review board number COA 047/2567) and was performed in accordance with the tenets of the Declaration of Helsinki. Informed consent was waived from all patients because of the retrospective nature of the fully anonymized radiographic images. The radiographic images were accessed on May 14, 2024 for the development of the Mask R-CNN model.

### Data preparation

Electronic health records of patients aged 18 years or older with routine root canal treatment history were retrieved from the endodontic clinic at Thammasat University hospital for a period from January 2015 to June 2021. The cases chosen for assessing treatment outcomes were selected from those categorized as having a low to moderate degree of endodontic treatment difficulty, as per the AAE Endodontic Case Difficulty Assessment Form and Guidelines (AAE Endodontic Case Difficulty Assessment Form and Guideline, 2022). All intraoperative procedures adhere to the endodontic treatment protocol at the University Hospital. The operators were board certified endodontists. The non-surgical endodontic clinical protocols followed the standard procedures of the Endodontic Clinic at Thammasat University hospital. All cases were strictly performed under rubber dam isolation, involving conservative access cavity preparation, cleaning and shaping with standardized endodontic instruments, and irrigation with 2.5–5% sodium hypochlorite, saline, and 17% EDTA with ultrasonic activation. Obturation was done using consistent materials and techniques, specifically the warm vertical compaction technique with a resin sealer, ensuring a proper coronal seal. Cases with intraoperative and/or postoperative errors were excluded. Therefore, the accuracy of the results was assured. In evaluating endodontic treatment outcomes, the parameters include clinical and radiographic examinations, which must be synchronized to accurately classify cases. According to the American Association of Endodontists (AAE) and American Academy of Oral and Maxillofacial Radiology (AOMR) Joint Position Statement (2016), 2-D intraoral radiographs should be the imaging modality of choice for evaluating endodontic patients. CBCT should be considered only when conventional radiographs do not provide adequate information. In this study, cases that required CBCT imaging were excluded.

Three board certified endodontists reviewed the results of endodontically treated teeth over a 3-year follow-up period, assessing outcomes through both radiographic (periapical radiographs) and clinical measures. Employing criteria for clinical and radiographic evaluation, the three endodontists categorized the periapical radiographs from the 3-year follow-up into three groups: healed, healing, and disease. In assessing the outcome of endodontic treatment, we used the guidelines for clinical and radiographic assessment as stated by Friedman and Mor [1]. The criteria were as follows: Healed–No clinical signs or symptoms and radiographic evidence of normal periapical tissues; Healing–Reduced size of periapical radiolucency without clinical signs and symptoms; Disease–Presence of clinical signs or symptoms and/or radiographic evidence of periapical radiolucency.

Digital periapical radiographic images were obtained with equipment from different manufacturers using standard imaging protocols. The digital periapical radiographs were taken using the paralleling technique. Exposure settings were 60–70 kilovoltage peak (kVp), 4–15 milliamperage (mA), and an exposure time between 0.1 to 1.0 seconds, depending on the tooth site and patient size. The digital sensors used were Size 1 for anterior periapical images and Size 2 for posterior periapical images, with a resolution of 20 line pairs per mm. The Rinn XCP (Extension Cone Paralleling) system was used to hold the digital sensor. All healed and disease teeth were evaluated and included in this study. To overcome the non-distribution of datasets given the low number of healed and disease teeth, preoperative periapical radiographic images of 1200 teeth with 3-year follow-up results were included and divided into healed (440 teeth), healing (400 teeth) and disease (360 teeth).

**Fig 2:**
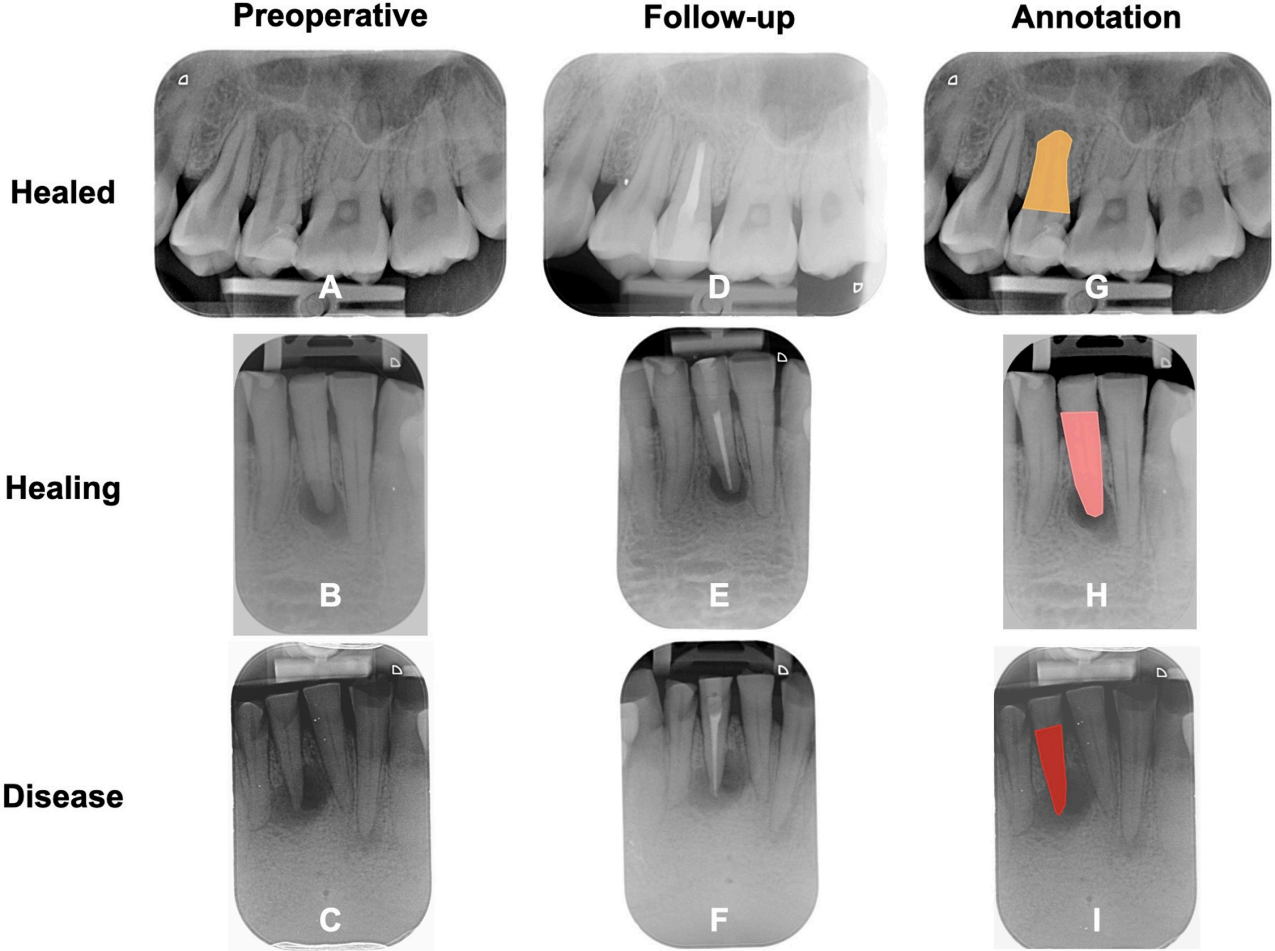
Examples of preoperative radiographic images (**A-C**); follow-up postoperative radiographic images (**D-F**); and polygon annotation on the preoperative radiographic images (**G-I**) of healed, healing and disease cases.

All preoperative periapical radiographic images were uploaded to the VisionMarker server and web application for image annotation. The public version is available on GitHub (GitHub, Inc., CA, USA). The tooth crown has various characteristics, such as different stages of tooth decay and different types of restoration materials. To reduce such confounding variables, this study focuses only on the part of the root in model development. Annotation is the process of outlining the root and identifying images to be classified into healed, healing or disease categories. The images were annotated by drawing the root area with polygon shape representing healed, healing and disease class (Fig 2). The root boundaries of the periapical radiographic images were annotated by three board certified endodontists. Owing to the differences in manual annotating from one endodontist to another, the ground truth used was the largest area of intersection between all of the endodontists’ annotations. A total of 1080-image dataset was used for model training, validation and testing. To avoid using the training images for further testing, the dataset was split into three parts: 80% training, 10% validation, and 10% testing. The training dataset was used for training the model while the validation dataset was independent of the training of the model. The model was tested on this dataset to stop training or revise training variables. The hold-out test dataset was used to test the trained model. An independent 120-image dataset was used for clinician evaluation.

### Deep learning model

This work applied a segmentation algorithm focusing on the root status for the prediction of the treatment outcome class. Segmentation is a fundamental task in image processing that involves dividing an image into meaningful segments. Mask Region-based Convolutional Neural Network (Mask R-CNN), an extension of the Faster R-CNN object detection algorithm, was used in this study. Mask R-CNN is a powerful deep learning model which combines object detection and instance segmentation [15].

**Fig 3:**
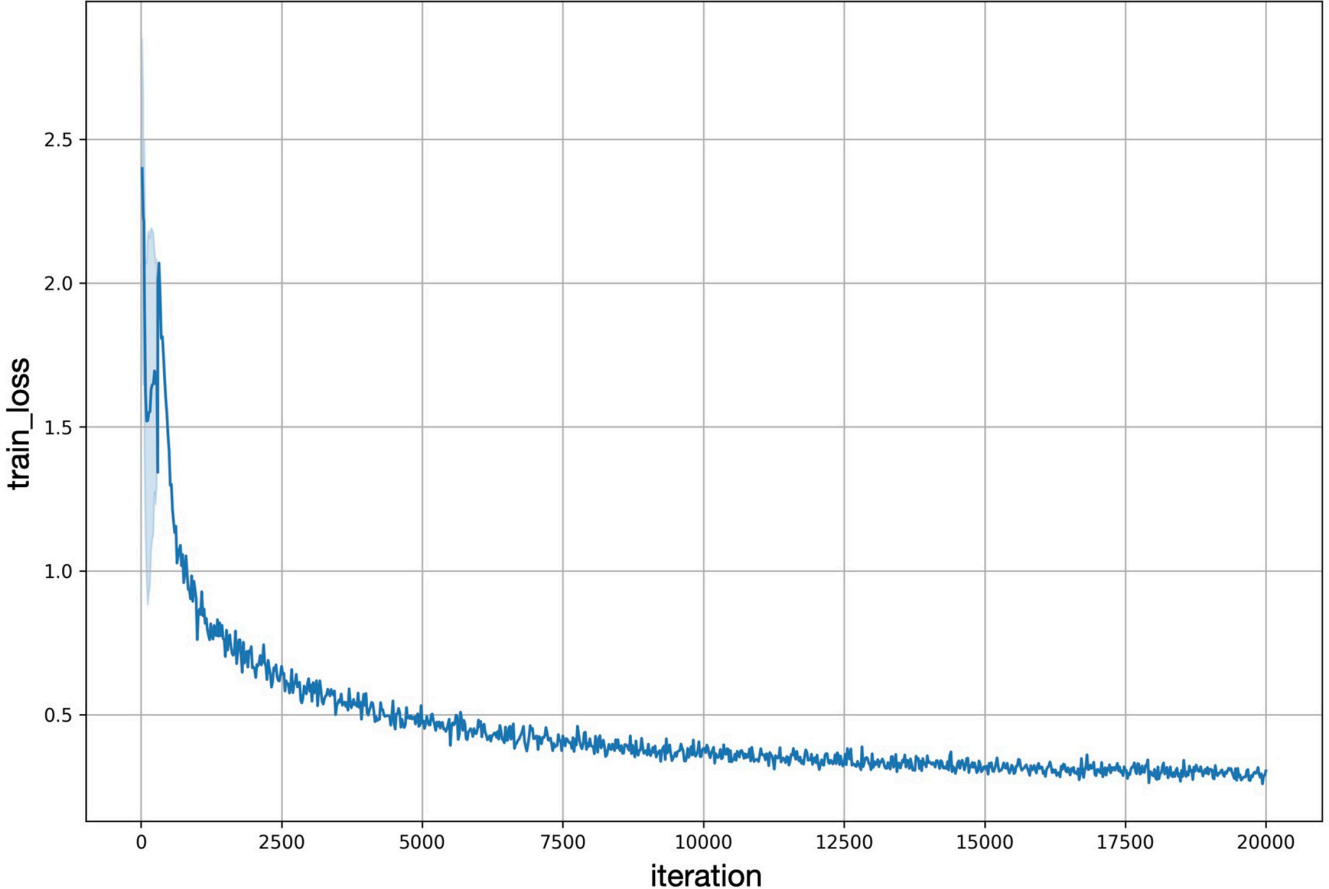
Mask R-CNN training loss graph. The x-axis represents training iterations or epochs, and the y-axis represents the loss values. The reported scale and decreased to a value close to 0 indicates that the model has effectively learned from the training data, enabling it to recognize object shapes and make accurate classifications.

The images were pre-processed by augmentation using Keras Image Data Generator (open-source software). The framework then resized an input image to 256 × 256 pixels to feed into Mask R–CNN model. The model was pre-trained on ImageNet and COCO (common objects in common) datasets. The training was performed on an on-premises server with GPU, Nvidia Tesla V100 32GB vRAM (Nvidia Corporation), Nvidia Driver 470.82 (Nvidia Corporation), and CUDA 11.4 (Nvidia Corporation) for 20000 iterations, with 0.025 learning rate, 1882 epochs, and a batch size of 64 images on the training dataset of annotated radiographs. The training loss was reduced and maintained between 15000 and 20000 iterations (data in S1 File Mask R-CNN model development and annotation). The training loss graph of Mask R-CNN revealed that the reported scale and decreased to a value close to 0. This indicates that the model has effectively learned from the training data, enabling it to recognize object shapes and make accurate classifications (Fig 3).

**Fig 4:**
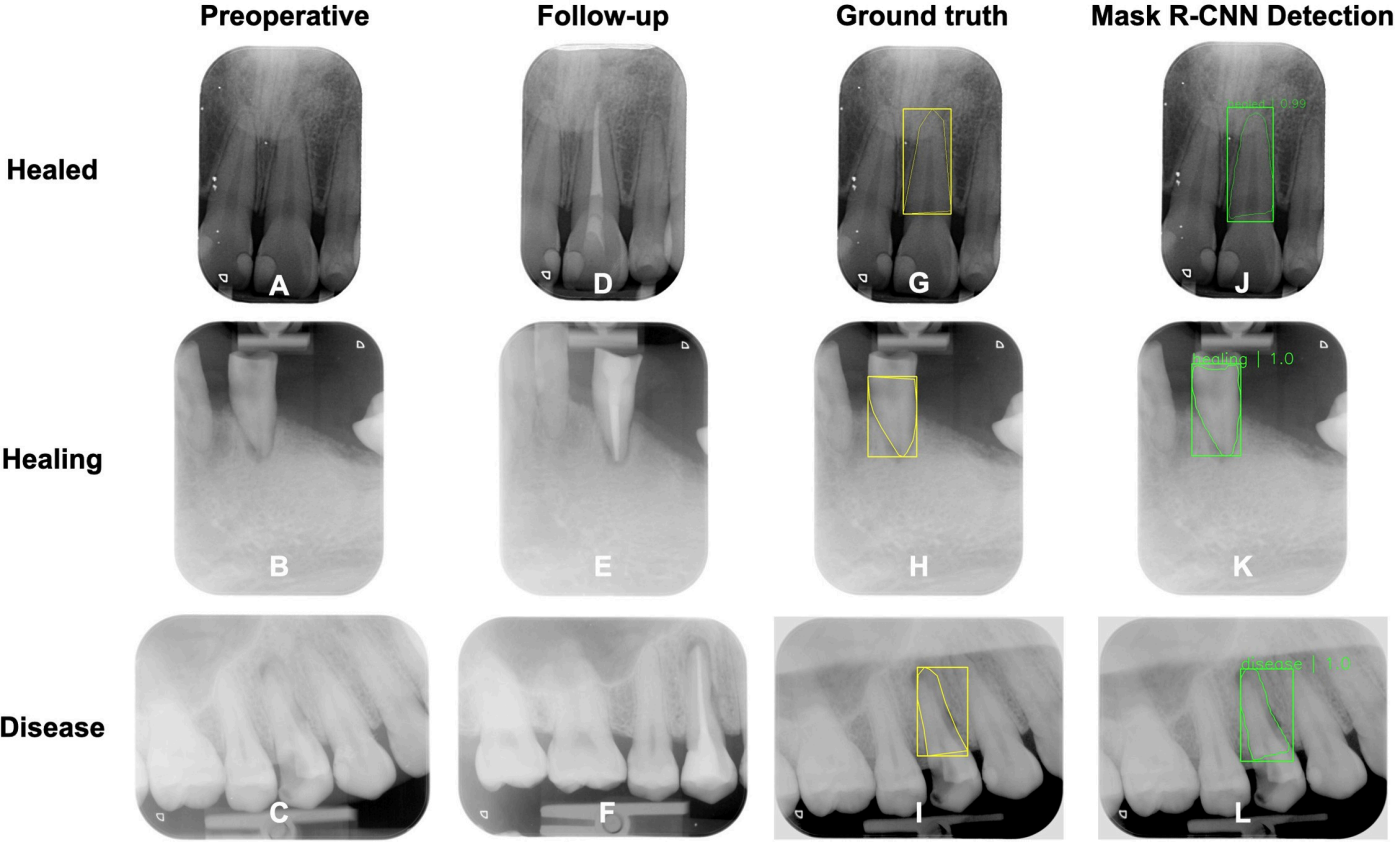
Preoperative radiographic images of healed, healing and disease **(A-C)**; 2-year follow-up postoperative radiographic images of healed, healing and disease **(D-F)**; Segmentation and bounding box ground truth based on endodontists’ annotations of the preoperative radiographic images of the healed, healing and disease **(G-I)**; The true positive outputs from Mask R-CNN segmentation model **(J-L)**.

In this study, Mask R–CNN used the annotated preoperative periapical radiographic images with known treatment outcome to segment the root area by learning from each pixels from the ground truth images. After the positions and shapes of the root were determined, predicting the treatment outcome class was performed. The treatment outcome probabilities are shown next to the bounding boxes of the mask area (Fig 4). The image in the Fig includes root masks in addition to bounding boxes and matching scores. The performance of the segmentation model was evaluated using 10% testing dataset to detect a segmentation with bounding box relative to the ground truth region in the healed, healing and disease images.

### Clinician evaluation

An independent 120-image dataset with known treatment results (healed ‐ 40 teeth, healing ‐ 40 teeth, disease ‐ 40 teeth) was evaluated to compare the performance of the Mask R-CNN prediction model with that of 20 clinicians; 10 experts who are board certified endodontists and 10 general partitioners (GPs) who have at least 2 years of experience in endodontic practice. None of these readers participated in the clinical care or assessment of the enrolled patients, nor did they have access to their medical records. All clinicians each independently evaluated preoperative periapical radiographic image of these 120 teeth manually and reevaluated them with the assist of Mask R-CNN prediction. For each tooth, the clinicians verified whether the prediction result generated by Mask R-CNN matched their personal evaluation. If there was a discrepancy, the clinicians made the final judgment based on their own clinical experience, taking into account the machine-generated result. After a 1-month interval, they evaluated the same images again in a shuffled order.

### Statistical analysis

The data analyses were conducted using IBM SPSS Statistics version 22.0 (IBM Corp., Armonk, NY, USA). The performance of the segmentation model was evaluated using 10% testing dataset to detect a segmentation with bounding box relative to the ground truth region in the healed, healing and disease images by the following matrices [16]:

1. Precision: the accuracy of the model’s positive predictions calculated by the ratio of true positives (correctly predicted objects) to the total number of positive predictions made by the model.
2. Recall (sensitivity): the ability of the model to find all positive instances calculated by the ratio of true positives to the total number of actual positive instances in the dataset.
3. F1 score: the harmonic mean of precision and recall providing a single metric that balances the trade-off between false positives and false negatives.
4. Area under the precision-recall curve (AUC): created by plotting precision (positive predictive value) against recall (true positive rate) at various classification thresholds.
5. Mean average precision (mAP): a single scalar that summarizes the accuracy of object segmentation across multiple object classes.

Segmentation accuracy was measured with the intersection over union (IoU) metric between segmentation with bounding box detection and ground truth, and was calculated by a pairwise IoU operation in Detectron. If the IoU value between the generated segmentation with bounding box and the ground truth was less than 0.5, then the produced segmentation with bounding box was considered to be a false detection. The statistical analysis for segmentation algorithm was calculated as follows:

IoU = area of overlap / area of union,      (1)
Precision = TP/TP + FP,        (2)
Recall (Sensitivity) = TP/TP + FN,       (3)
F1 Score = 2 (Precision x Recall) / (Precision + Recall).  (4)

True positive (TP) is positive outcomes that the model predicted correctly, in which IoU > 0.5. False positive (FP) is positive outcomes that the model predicted incorrectly, in which IoU < 0.5. False negative (FN) is negative outcomes that the model predicted incorrectly. mAP is the mean Average Precision of all classes. 95% confidence intervals (CI) were calculated in evaluating these metrics.

In clinician evaluation, the average sensitivity and specificity, as well as the mAP of predicting endodontic treatment outcomes from preoperative periapical radiographs with and without the help of the Mask R-CNN model were calculated. The intra-rater reliability analysis of each endodontist and GP, as well as the inter-rater reliability analysis of the endodontist group and GP group, were calculated by Cohen’s kappa [17]. The intra-rater and inter-rater reliability analysis was interpreted using the benchmark thresholds proposed by Landis and Koch [18], with Cohen’s kappa ≥ 0.80 representing excellent agreement.

## Results

A total of 1,200 cases (46.3% male and 53.7% female; 79.8% were 25–64 years old) treated by board certified endodontists were included in this study (Table 1). The majority of cases were primary endodontic treatments with pulpal diagnoses of asymptomatic irreversible pulpitis (34.3%), symptomatic irreversible pulpitis (28.2%), and necrosis (32.2%). The periapical diagnoses included normal (4.5%), apical periodontitis (81.9%), chronic apical abscess (12.7%), acute apical abscess (0.3%), and other conditions (e.g., condensing osteitis 0.6%). The types of final restorations included direct composite restoration (4.3%), inlay or onlay (2.1%), crown (26.7%), post and core with crown (66.2%), and endocrown (0.7%).

**Table 1:** Demographic and clinical data.

Attribute	Attribute value	Frequency (*n* = 1200)
Gender	Male	556 (46.3%)
	Female	644 (53.7%)
Age	Less than 24	176 (14.7%)
	25–64	958 (79.8%)
	More than 65	66 (5.5%)
Pulpal diagnosis	Normal	0
	Asymptomatic irreversible pulpitis	412 (34.3%)
	Symptomatic irreversible pulpitis	338 (28.2%)
	Necrosis	387 (32.2%)
	Previously initiated therapy	38 (3.2%)
	Previously root canal treatment	25 (2.1%)
Periapical diagnosis	Normal	54 (4.5%)
	Asymptomatic apical periodontitis	487 (40.6%)
	Symptomatic apical periodontitis	495 (41.3%)
	Chronic apical abscess	153 (12.7%)
	Acute apical abcess	4 (0.3%)
	Other	7 (0.6%)
Type of final restoration	Direct composite restoration	52 (4.3%)
	Inlay or onlay	25 (2.1%)
	Crown	321 (26.7%)
	Post and core with crown	794 (66.2%)
	Endocrown	8 (0.7%)

### Performance of Mask R-CNN model

**Fig 5:**
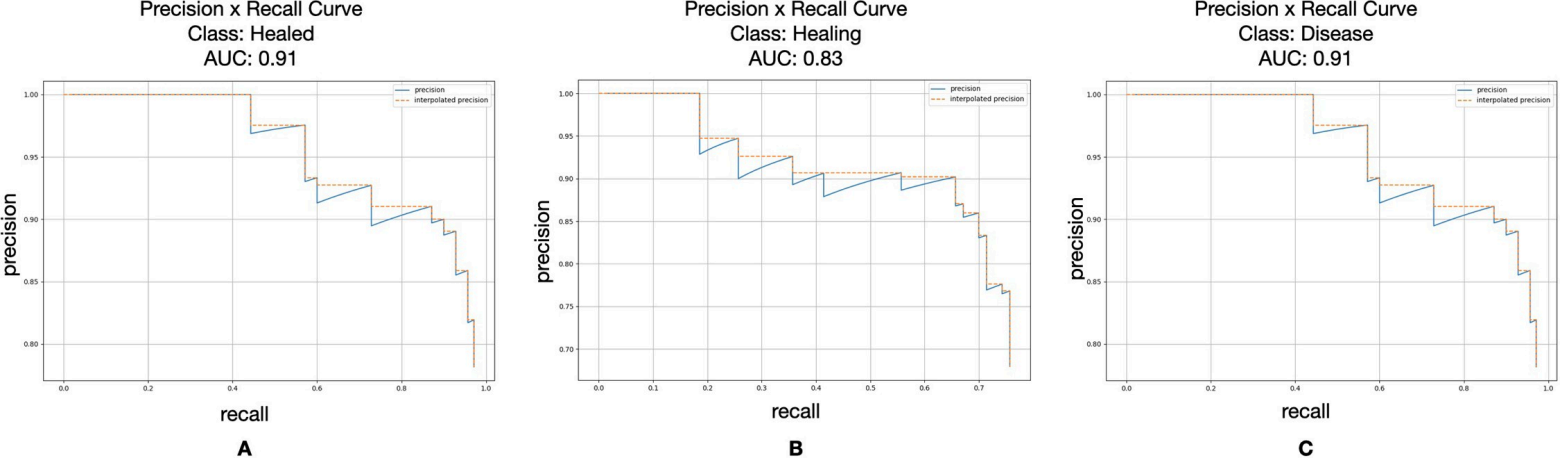
The precision-recall curve for multiclass segmentation of Mask R-CNN. The area under the precision-recall curve (AUC) were 0.91, 0.83 and 0.91 for prediction of healed (**A**), healing (**B**), and disease (**C**).

The deep learning-based endodontic treatment outcome prediction model was evaluated on the test set and the results are reported in Table 2. The segmentation and class prediction performance of Mask R-CNN segmentation model achieved high precision, recall, F1 score and AUC of precision-recall curve. The mAP of Mask R-CNN was 0.88 (95% CI 0.83–0.93). The overall prediction performance of endodontic treatment outcome with AUC of precision-recall was 0.91 (95% CI 0.88–0.94), 0.83 (95% CI 0.81–0.85), 0.91 (95% CI 0.90–0.92) on healed, healing and disease, respectively (Fig 5). An AUC of 1.0 indicates perfect prediction performance. An AUC of 0.5 suggests random prediction performance (equivalent to chance). AUC values between 0.5 and 1.0 indicate varying degrees of prediction accuracy above chance. Therefore, in our study: AUC of 0.91 for healed indicates a high accuracy in predicting healed outcomes; AUC of 0.83 for healing indicates a moderate accuracy in predicting healing outcomes; AUC of 0.91 for disease indicates a high accuracy in predicting disease outcomes. Examples of segmentation and class prediction outputs from Mask R-CNN segmentation model in this study are provided in Fig 4.

**Table 2:** Multi-class segmentation performances of Mask R-CNN algorithms on a test dataset.

	Class	Precision	Recall (Sensitivity)	F1 score	AUC of precision-recall curve	mAP
Mask R-CNN	Healed	0.88 (0.85–0.91)	0.92 (0.89–0.94)	0.90 (0.84–0.96)	0.91 (0.88–0.94)	0.88 (0.83–0.93)
	Healing	0.84 (0.78–0.90)	0.86 (0.79–0.92)	0.82 (0.79–0.85)	0.83 (0.81–0.85)	
	Disease	0.92 (0.90–0.94)	0.88 (0.85–0.91)	0.90 (0.84–0.96)	0.91 (0.90–0.92)	

AUC, area under the receiver operating characteristics curve

Metrics uses average value (95% CI)

### Comparison with clinician performance

To thoroughly assess the applicability of Mask R-CNN model, we conducted a comprehensive comparison of its performance with clinicians for predicting endodontic treatment outcome on preoperative periapical radiographs. Results of the clinician prediction with and without the help of Mask R-CNN are shown in Table 3. The prediction metrics of general practitioners and endodontists significantly improved with the help of Mask R-CNN outperforming clinicians alone with mAP increasing from 0.75 (0.72–0.78) to 0.84 (0.81–0.87) and 0.88 (0.85–0.91) to 0.92 (0.89–0.95), respectively. The intra-rater reliability of each GP and endodontist showed excellent agreement (Cohen’s kappa ranging from 0.87 to 0.95). Regarding inter-rater reliability, both the GP group (Cohen’s kappa of 0.81) and the endodontist group (Cohen’s kappa of 0.85) reached excellent agreement.

**Table 3:** The performance of clinicians (GPs vs endodontists) with and without the help of Mask R-CNN for endodontic treatment outcome prediction from preoperative radiographic images on an independent dataset.

		First time			Second time			
	Class	Sensitivity	Specificity	Precision	Sensitivity	Specificity	Precision	mAP
GPs	Healed	0.79 (0.76–0.82)	0.80 (0.76–0.84)	0.78 (0.74–0.82)	0.75 (0.72–0.77)	0.78 (0.74–0.82)	0.76 (0.73–0.79)	0.75 (0.72–0.78)
	Healing	0.71 (0.70–0.72)	0.73 (0.70–0.76)	0.71 (0.69–0.73)	0.72 (0.70–0.74)	0.71 (0.68–0.74)	0.70 (0.69–0.71)	
	Disease	0.78 (0.76–0.80)	0.77 (0.74–0.80)	0.76 (0.73–0.79)	0.79 (0.75–0.83)	0.80 (0.75–0.85)	0.79 (0.74–0.84)	
GPs with Mask R-CNN	Healed	0.85 (0.83–0.87)	0.87 (0.81–0.93)	0.86 (0.81–0.91)	0.86 (0.81–0.91)	0.90 (0.87–0.93)	0.88 (0.85–0.91)	0.84 (0.81–0.87)
	Healing	0.80 (0.75–0.85)	0.81 (0.78–0.84)	0.80 (0.76–0.84)	0.77 (0.73–0.81)	0.79 (0.73–0.85)	0.78 (0.76–0.80)	
	Disease	0.83 (0.80–0.86)	0.87 (0.85–0.89)	0.85 (0.83–0.87)	0.86 (0.85–0.87)	0.87 (0.81–0.93)	0.87 (0.82–0.92)	
Endodontists	Healed	0.91 (0.89–0.94)	0.89 (0.85–0.93)	0.89 (0.84–0.94)	0.92 (0.90–0.94)	0.89 (0.86–0.92)	0.90 (0.86–0.94)	0.88 (0.85–0.91)
	Healing	0.86 (0.81–0.91)	0.84 (0.81–0.87)	0.85 (0.83–0.87)	0.85 (0.83–0.87)	0.89 (0.84–0.94)	0.87 (0.83–0.91)	
	Disease	0.88 (0.85–0.91)	0.87 (0.83–0.91)	0.88 (0.85–0.91)	0.89 (0.85–0.93)	0.88 (0.84–0.92)	0.89 (0.84–0.94)	
Endodontists with Mask R-CNN	Healed	0.92 (0.90–0.94)	0.91 (0.88–0.94)	0.93 (0.90–0.96)	0.95 (0.91–0.99)	0.91 (0.89–0.93)	0.92 (0.89–0.95)	0.92 (0.89–0.95)
	Healing	0.88 (0.83–0.95)	0.91 (0.89–0.93)	0.89 (0.85–0.93)	0.92 (0.90–0.94)	0.89 (0.86–0.92)	0.90 (0.89–0.91)	
	Disease	0.95 (0.92–0.98)	0.92 (0.89–0.95)	0.94 (0.89–0.99)	0.95 (0.92–0.98)	0.93 (0.90–0.96)	0.94 (0.90–0.98)	

GP, general practitioner

Metrics uses average value (95% CI)

## Discussion

Mask R-CNN is designed to perform instance segmentation, which involves not only object detection but also pixel-wise segmentation of objects within an image [15]. This capability makes it particularly useful in medical and dental applications where precise object localization and segmentation are crucial. Mask R-CNN has been used to identify and segment tumors in medical images such as ultrasound images [19]. This work was valuable in classifying the benign or malignant nature of breast nodules. Medical professionals use Mask R-CNN to segment and identify specific organs or structures within the body, which is essential for surgical planning and image-guided interventions [20]. In dentistry, tooth segmentation and numbering were performed using Mask R–CNN on bitewing radiographic images. High quality segmentation masks were obtained in addition to the bounding box and class scores compared to other convolutional neural networks [21]. The model can be used to identify and segment dental caries in radiographic images, helping with the early detection and treatment of dental caries [22].

From our knowledge, this study was the first to implement Mask R-CNN to predict non-surgical endodontic treatment outcomes. Unlike classification or object detection that uses the entire radiographic image or root bounding box, the segmentation algorithm was selected because it separated the root, area of interest, from the surrounding structures to train the prediction model. The results of this study demonstrated the high performance with mean average precision of 0.88 of a deep learning-based Mask R-CNN for predicting endodontic treatment outcomes via root segmentation in preoperative radiographic images. There is room for enhancement, and we aspire to achieve this in the future. This could involve incorporating more input data to further train the model and adopting more advanced, accurate deep learning technologies as they emerge. Prediction performance was highest for ‘disease’ class followed by ‘healed’ class, and the lowest prediction performance was for ‘healing’ class, as shown by the area under the precision-recall curve of 0.91, 0.91 and 0.83 respectively. The category ‘healing’ received the least prediction score. This finding related to the clinician evaluation. With the assist of the Mask R-CNN prediction model, GPs and endodontists achieved superior metrics in prediction of endodontic treatment outcome from periapical radiographs. This study confirmed our hypothesis that integrating the Mask R-CNN model with clinicians would improve the accuracy of predicting endodontic treatment outcomes on preoperative periapical radiographs compared to predictions made by clinicians alone. We demonstrated a significant improvement in predictive performance when clinicians used the Mask R-CNN model alongside their own assessments. Specifically, the mean Average Precision (mAP) increased from 0.75 to 0.84 for general practitioners and from 0.88 to 0.92 for endodontists. These results suggest that integrating AI technology can enhance the diagnostic accuracy of clinicians in endodontic practice, potentially leading to improved treatment planning and patient outcomes.

The results of this study align with previous research on the automatic detection of dental caries in periapical radiographs using convolutional neural network architecture [23]. Artificial intelligence technology is increasingly being applied in endodontics. Studies on AI applications in endodontics have shown that AI can enhance diagnosis and treatment, leading to improved endodontic treatment outcomes [10]. Numerous studies have demonstrated the effectiveness of deep learning applications in endodontics, including the identification of periapical lesions [24] and root fractures [25], investigation of root canal system anatomy, and assessment of working lengths [11], detection of separated root canal instruments [26], and integration of tooth and root detection to improve surgical planning [27]. These results suggest that such applications may benefit beginners and non-specialists by providing expert judgment and clinical decision support.

In this study, all endodontic cases were selected based on the criteria of clinical and radiographic outcomes [9]. The presence or absence of periapical lesions was one of several factors assessed during the classification of treatment outcomes. Although, three-dimensional (3D) imaging has become increasingly important in the field of endodontics for diagnosis and treatment planning, providing a more detailed and accurate understanding of tooth anatomy and pathology [28]. However, in this study, 2D periapical radiographs were considered as the ground truth because 2D radiography remains the routine choice for most clinicians and endodontists.

Our work on developing a high-performance Mask R-CNN model for classifying endodontic treatment outcomes has significant implications for endocontic treatment planning. By providing accurate and reliable classifications of treatment outcomes (healed, healing, and disease), the model can assist clinicians in making more informed decisions regarding the necessity and type of further interventions. This precision can lead to optimized treatment plans tailored to individual patient needs, potentially reducing the incidence of unnecessary procedures and improving overall treatment efficiency. The model serves as a decision-support tool, augmenting the clinician diagnostic capabilities and potentially reducing the cognitive load and uncertainty associated with assessing treatment outcomes. This can be particularly beneficial for less experienced practitioners or those dealing with complex cases. The results of applying Mask R-CNN model in endodontics can inspire further studies on its application in other dental specialties. It sets a precedent for the use of deep learning models in clinical diagnostics, encouraging researchers to develop, refine, and validate similar technologies.

There were several limitations to our work. First, the preoperative radiographic image data used for the experiments were retrospective data from a single hospital, involving cases with a low to moderate degree of difficulty. This potentially limits the generalizability of the prediction model. Second, we only included preoperative periapical radiographic images, omitting other important preoperative patient history, signs, and symptoms that should be included in the model. Lastly, compared to large-scale medical imaging datasets, our dataset was extremely small. Algorithm development could benefit from more data from other hospitals or institutions, which would provide more categories and lead to better performance. For future work, the multicenter collection of preoperative radiographic image data of all difficulty categories and the inclusion of intraoperative and postoperative complications with the integration of AI algorithms for image analysis and cognitive analysis should enable the generalization of the use of the prediction model in clinical decision-making. Integrating AI into clinical applications can be difficult due to clinicians’ distrust of computer predictions and the potential risks associated with erroneous results [29]. Future work should be designed to use AI models to trigger a second opinion in cases of disagreement between the clinician and the algorithm. By keeping AI predictions hidden throughout the diagnostic process, the risks associated with distrust and incorrect predictions could be minimized, relying solely on human predictions.

## Conclusions

Under the conditions of this study, the deep learning-based Mask R-CNN model demonstrated high performance in classifying endodontic treatment outcomes into healed, healing, and disease categories using preoperative periapical radiographic images. The accuracy of clinicians in assessing non-surgical endodontic treatment outcomes was improved when assisted by the Mask R-CNN model compared to their assessments alone. This model is expected to aid in endodontic treatment planning.

## Supporting information

S1 File Mask R-CNN model development and annotation. (DOCX)
