# Supplementary material for: Development and evaluation of a deep learning segmentation model for assessing non-surgical endodontic treatment outcomes on periapical radiographs: A retrospective study
Source: PLoS One. 2024 Dec 31;19(12):e0310925. doi: 10.1371/journal.pone.0310925 (PMC11687807; doi:10.1371/journal.pone.0310925)

**Mask R-CNN model development and annotation**

*# Download annotation images from labelstudio*

In [1]:

**import** os

**import** json

**import** requests

**from** tqdm.auto **import** tqdm

**import** urllib

**import** cv2

**import** numpy **as** np

**import** threading

**import** math

In [2]:

host **=** "192.168.24.160:8082"

token **=** "e764506728e98e51b5659d386757ca83ca8744c5"

projects **=** {

7: 'healed',

6: 'healing',

5: 'disease'

}

base_dir **=** 'dataset'

os**.**makedirs(base_dir, exist_ok**=True**)

In [3]:

annotations **=** {}

**for** project_id, project_name **in** projects**.**items():

data **=** requests**.**get(f"http://{host}/api/projects/{project_id}/export?exportType=JSON",

headers**=**{

'Authorization': f"Token {token}"

}

)

annotation **=** data**.**json()

[elm**.**update({'class': project_name}) **for** elm **in** annotation]

annotations[project_name] **=** annotation

**with** open(f'dataset/annotations-{project_name}.json', 'w') **as** f:

json**.**dump(annotations, f)

print(f"Saved annotations {project_name} => {len(annotation)}")

Saved annotations healed => 396

Saved annotations healing => 360

Saved annotations disease => 324

In [4]:

datasets **=** []

**for** dataset_name, dataset **in** annotations**.**items():

datasets **+=** dataset

**with** open(f'dataset/annotations.json', 'w') **as** f:

json**.**dump(datasets, f)

print(f"number of dataset : {len(datasets)}")

number of dataset : 1080

In [5]:

**def** splitList(data, num, num_threads**=**8):

window **=** len(data)**/**num_threads

start **=** math**.**ceil(num ***** window)

stop **=** math**.**ceil((num**+**1) ***** window)

**return** data[start:stop]

**def** get_image(thread_no, dataset):

**for** item **in** tqdm(dataset):

data_path **=** item**.**get('data', {})**.**get('image')

file_name **=** data_path**.**split('/')[**-**1]

req **=** urllib**.**request**.**Request(f'http://{host}{data_path}')

req**.**add_header('Authorization', f"Token {token}")

req **=** urllib**.**request**.**urlopen(req)

arr **=** np**.**asarray(bytearray(req**.**read()), dtype**=**np**.**uint8)

frame **=** cv2**.**imdecode(arr, **-**1)

image_path **=** os**.**path**.**join(base_dir, 'images', file_name)

cv2**.**imwrite(image_path, frame)

In [6]:

NUM_THREAD **=** 12

os**.**makedirs(os**.**path**.**join(base_dir, 'images'), exist_ok**=True**)

threads**=**[]

**for** i **in** range(NUM_THREAD):

print(f"Main : create and start thread {i}.")

blukData **=** splitList(datasets, i, num_threads**=**NUM_THREAD)

thread **=** threading**.**Thread(target**=**get_image, args**=**(i,blukData, ) )

threads**.**append(thread)

thread**.**start()

**for** index, thread **in** enumerate(threads):

print(f"Main : before joining thread {index}.")

thread**.**join()

print(f"Main : thread {index} done")

*# Manage the notation for train test and validation split*

**import** os

**import** glob

**import** json

**import** shutil

**import** pandas **as** pd

**import** math

**from** sklearn.model_selection **import** train_test_split

In [7]:

base_dir **=** 'dataset'

In [8]:

ls dataset

annotations-disease.json annotations.json **images**/

annotations-healed.json count-dataset.csv

annotations-healing.json dataset-healed.json

In [9]:

df **=** pd**.**read_json(os**.**path**.**join(base_dir, 'annotations.json'))

df **=** df**.**astype({

'created_at': 'string',

'updated_at': 'string'

})

df **=** df[['id', 'class', 'annotations', 'file_upload', 'data', 'created_at', 'updated_at', 'project']]

df**.**head(3)

In [10]:

df**.**loc[0, 'annotations']

Out[10]:

[{'id': 3141,

'completed_by': 3,

'result': [{'id': 'p3_iMF36X3',

'type': 'polygonlabels',

'value': {'closed': True,

'points': [[7.08502024291498, 66.7185069984448],

[10.931174089068826, 57.85381026438569],

[15.182186234817813, 52.41057542768274],

[17.408906882591094, 47.744945567651634],

[19.635627530364374, 44.32348367029549],

[20.850202429149796, 41.990668740279936],

[23.279352226720647, 43.07931570762053],

[25.708502024291498, 45.41213063763608],

[25.910931174089068, 49.61119751166407],

[23.279352226720647, 68.58475894245723]],

'polygonlabels': ['healed']},

'origin': 'manual',

'to_name': 'image',

'from_name': 'label',

'image_rotation': 0,

'original_width': 1484,

'original_height': 1930}],

'was_cancelled': False,

'ground_truth': False,

'created_at': '2023-06-01T09:55:43.477058Z',

'updated_at': '2023-06-01T09:55:43.477085Z',

'lead_time': 27.668,

'prediction': {},

'result_count': 0,

'unique_id': '7e8d3303-e8c6-4dc5-9738-6a9e68581ba7',

'last_action': None,

'task': 3072,

'project': 7,

'updated_by': 3,

'parent_prediction': None,

'parent_annotation': None,

'last_created_by': None}]

In [11]:

classes **=** df['class']**.**unique()

classes

Out[11]:

array(['healed', 'healing', 'disease'], dtype=object)

In [12]:

dataset_splits **=** {dataset: [] **for** dataset **in** ['train', 'test', 'validate']}

**for** cls **in** classes:

tmp_df **=** df**.**copy()

tmp_data **=** list(tmp_df[tmp_df['class']**==**cls]**.**T**.**to_dict()**.**values())

Train, Test **=** train_test_split(tmp_data, test_size**=**0.2, random_state**=**1)

Train, Validate **=** train_test_split(Train, test_size**=**0.125, random_state**=**1)

[elm**.**update({'dataset': 'train'}) **for** elm **in** Train]

[elm**.**update({'dataset': 'test'}) **for** elm **in** Test]

[elm**.**update({'dataset': 'validate'}) **for** elm **in** Validate]

dataset_splits['train'] **+=** Train

dataset_splits['test'] **+=** Test

dataset_splits['validate'] **+=** Validate

In [13]:

df_count **=** pd**.**concat([pd**.**DataFrame(elm) **for** dataset, elm **in** dataset_splits**.**items()])\

**.**groupby(['dataset', 'class'])\

**.**agg({'id': 'count'})\

**.**rename(columns**=**{'id': 'numberOfDataset'})

df_count

Out[13]:

|  |  | **numberOfDataset** |
| --- | --- | --- |
| **dataset** | **class** |  |
| **test** | **disease** | 32 |
|  | **healed** | 40 |
|  | **healing** | 36 |
| **train** | **disease** | 260 |
|  | **healed** | 316 |
|  | **healing** | 288 |
| **validate** | **disease** | 32 |
|  | **healed** | 40 |
|  | **healing** | 36 |

df_count**.**reset_index()**.**to_csv(os**.**path**.**join(base_dir, 'count-dataset.csv'), index**=False**)

In [14]:

classes **=** list(df_count**.**reset_index()['class']**.**unique())

classes

Out[14]:

['disease', 'healed', 'healing']

In [15]:

**with** open(os**.**path**.**join(base_dir, 'dataset-meta.json'), 'w') **as** f:

data **=** {

'class': classes

}

json**.**dump(data, f)

In [16]:

dataset_splits**.**keys()

Out[16]:

dict_keys(['train', 'test', 'validate'])

In [17]:

**for** dataset_name, data **in** dataset_splits**.**items():

**with** open(os**.**path**.**join(base_dir ,f"dataset-{dataset_name}.json"), 'w') **as** f:

json**.**dump(data, f)

**3. training mask rcnn model**

*# pip install imgaug*

In [18]:

**import** os

**import** json

**import** cv2

**import** numpy **as** np

**import** pandas **as** pd

**import** pickle

**from** tqdm.notebook **import** tqdm

**import** random

**import** matplotlib.pyplot **as** plt

**import** math

**from** urllib.parse **import** quote_plus

**from** imgaug.augmentables.bbs **import** BoundingBox

**from** imgaug.augmentables.polys **import** Polygon

In [19]:

base_dir **=** 'dataset'

In [20]:

ls dataset

annotations-disease.json count-dataset.csv dataset-meta.json **images**/

annotations-healed.json dataset-disease.json dataset-test.json

annotations-healing.json dataset-healed.json dataset-train.json

annotations.json dataset-healing.json dataset-validate.json

In [21]:

df_datasets **=** {}

**for** dataset **in** ['train', 'validate', 'test']:

df_datasets[dataset] **=** pd**.**read_json(os**.**path**.**join(base_dir, f"dataset-{dataset}.json"))

df_datasets**.**keys()

Out[21]:

dict_keys(['train', 'validate', 'test'])

In [22]:

**with** open(os**.**path**.**join(base_dir, 'dataset-meta.json'), 'r') **as** f:

meta **=** json**.**load(f)

meta

Out[22]:

{'class': ['disease', 'healed', 'healing']}

In [23]:

**def** _get_bbox(points, width, height):

points **=** np**.**array(points)**/**100

X **=** points[:,0]

Y **=** points[:,1]

min_x, max_x **=** X**.**min()*****width, X**.**max()*****width

min_y, max_y **=** Y**.**min()*****height, Y**.**max()*****height

**return** [min_x, min_y, max_x, max_y]

**def** _convert_points(points, width, height):

points **=** np**.**array(points)**/**100

points[:,0] **=** points[:,0] ***** width

points[:,1] **=** points[:,1] ***** height

**return** points**.**tolist()

**def** _get_obj(annotation):

points **=** annotation**.**get('value', {})**.**get('points')

img_w, img_h **=** annotation**.**get('original_width'), annotation**.**get('original_height')

labels **=** annotation**.**get('value',{})**.**get('polygonlabels')

label **=** labels[0] **if** len(labels) **else** "-"

obj **=** {

"bbox": _get_bbox(points, img_w, img_h),

"bbox_mode": 0,

"segmentation": _convert_points(points, img_w, img_h),

"category_id": meta**.**get('class')**.**index(label) **if** label **in** meta**.**get('class') **else** **-**1

}

**return** obj

**def** _get_annotations(annotations):

segmentations **=** [elm**.**get('result', []) **for** elm **in** annotations]

segmentations **=** [item **for** sublist **in** segmentations **for** item **in** sublist]

data **=** [_get_obj(elm) **for** elm **in** segmentations]

**return** data

**def** get_annotations(row):

record **=** {

'image_id': row['id'],

'image_path': os**.**path**.**join(base_dir, 'images', quote_plus(row['file_upload'])),

'annotations': _get_annotations(row['annotations'])

}

height, width **=** cv2**.**imread(record**.**get('image_path'))**.**shape[:2]

record**.**update({

'file_name': record['image_path'],

'height': height,

'width': width

})

**return** record

In [24]:

**for** dataset, df **in** df_datasets**.**items():

df['data-annatations'] **=** df**.**apply(**lambda** x: get_annotations(x), axis**=**1)

In [25]:

os**.**makedirs(os**.**path**.**join(base_dir, 'maskRcnn'), exist_ok**=True**)

**for** dataset, df **in** df_datasets**.**items():

**with** open(os**.**path**.**join(base_dir, 'maskRcnn', f'{dataset}.json'), 'w') **as** f:

json**.**dump(df['data-annatations']**.**to_list(), f)

In [26]:

**def** cv2plt(frame):

**return** cv2**.**cvtColor(frame, cv2**.**COLOR_BGR2RGB)

In [27]:

samples **=** random**.**sample((df_datasets['train']['data-annatations'])**.**to_list(), k**=**20)

**for** i **in** range(0, len(samples)**-**1, 5):

sample **=** samples[i:i**+**5]

fig, ax **=** plt**.**subplots(1, 5, figsize**=**(16,10))

**for** j,sample **in** enumerate(sample):

frame **=** cv2**.**imread(sample['image_path'])

h,w **=** frame**.**shape[:2]

t **=** math**.**sqrt((w*****h))**/**1000

ploygon_cor **=** []

**for** marker **in** sample['annotations']:

x1,y1,x2,y2 **=** tuple(marker['bbox'])

bbox **=** BoundingBox(x1**=**x1, y1**=**y1, x2**=**x2, y2**=**y2)

frame **=** bbox**.**draw_on_image(frame, alpha**=**1, size**=**int(10*****t))

poly **=** Polygon(marker['segmentation'])

frame **=** poly**.**draw_on_image(frame, alpha**=**1, size**=**int(10*****t))

ax[j]**.**imshow(cv2plt(frame))

plt**.**show()


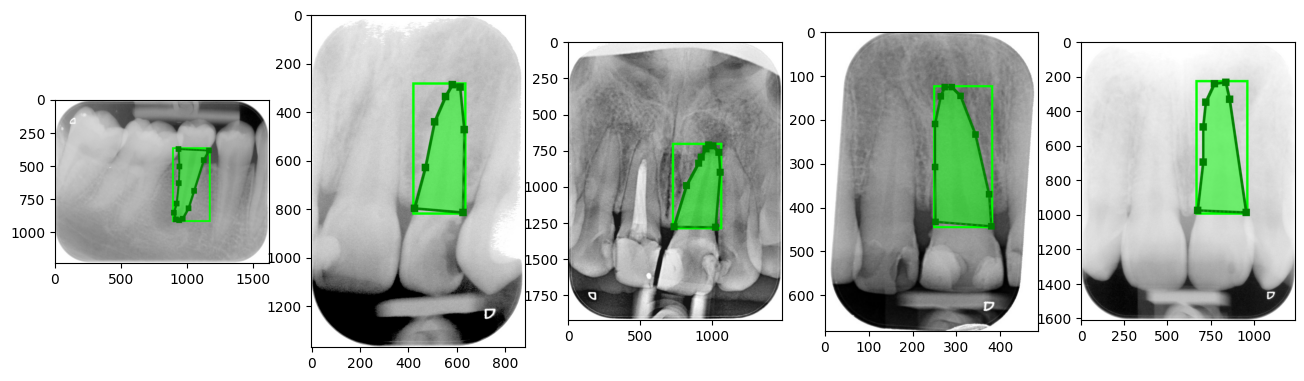

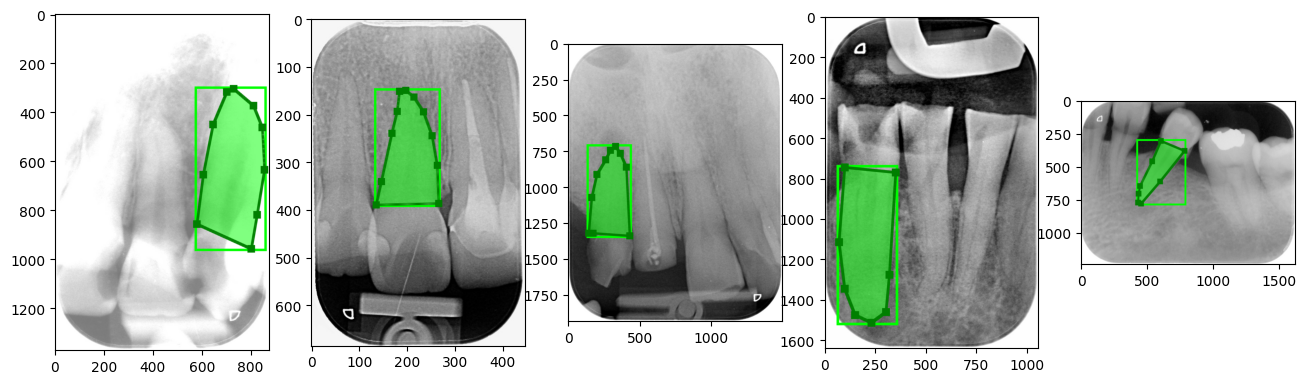
In [28]:

*# pip uninstall -y detectron2*

*# pip install detectron2 -f https://dl.fbaipublicfiles.com/detectron2/wheels/cu102/torch1.10/index.html*

*# pip install Pillow==9.0.1 setuptools==59.5.0*

In [29]:

**import** torch**,** torchvision

print(torch**.**__version__, torch**.**cuda**.**is_available())

1.10.0+cu102 True

In [30]:

*# Some basic setup:*

*# Setup detectron2 logger*

**import** detectron2

**from** detectron2.utils.logger **import** setup_logger

setup_logger()

*# import some common libraries*

**import** numpy **as** np

**import** os**,** json**,** cv2**,** random

**from** detectron2 **import** model_zoo

**from** detectron2.engine **import** DefaultPredictor

**from** detectron2.config **import** get_cfg

**from** detectron2.utils.visualizer **import** Visualizer

**from** detectron2.data **import** MetadataCatalog, DatasetCatalog

**import** matplotlib.pyplot **as** plt

**from** detectron2.structures **import** BoxMode

**import** pandas **as** pd

**import** seaborn **as** sns

In [31]:

base_dir **=** 'dataset'

In [32]:

**def** plot(frame):

plt**.**imshow(cv2**.**cvtColor(frame, cv2**.**COLOR_BGR2RGB))

plt**.**show()

**def** cv2plt(frame):

**return** cv2**.**cvtColor(frame, cv2**.**COLOR_BGR2RGB)

In [33]:

**import** json

**def** get_dicts(d):

**with** open(os**.**path**.**join(base_dir, 'maskRcnn',f'{d}.json'), 'r') **as** f:

data **=** json**.**load(f)

**for** i, item **in** enumerate(data):

**for** j, item2 **in** enumerate(item['annotations']):

data[i]['annotations'][j]['bbox_mode'] **=** BoxMode**.**XYXY_ABS

data[i]['annotations'][j]['segmentation'] **=** [[item **for** sublist **in** item2['segmentation'] **for** item **in** sublist]]

**return** data

In [34]:

*# get_dicts('train')[:3]*

In [35]:

**with** open(os**.**path**.**join(base_dir, 'dataset-meta.json'), 'r') **as** f:

meta **=** json**.**load(f)

meta

Out[35]:

{'class': ['disease', 'healed', 'healing']}

In [36]:

version **=** "79"

**try**:

MetadataCatalog**.**remove(f'oral_{version}_train')

DatasetCatalog**.**remove(f'oral_{version}_train')

MetadataCatalog**.**remove(f'oral_{version}_validate')

DatasetCatalog**.**remove(f'oral_{version}_validate')

**except** Exception:

**pass**

**for** d **in** ["train", "validate"]:

DatasetCatalog**.**register(f"oral_{version}_{d}", **lambda** d**=**d: get_dicts(d))

MetadataCatalog**.**get(f"oral_{version}_{d}")**.**set(thing_classes**=**meta**.**get('class',[]))

metadata **=** MetadataCatalog**.**get("oral_{}_train"**.**format(version))

In [37]:

dataset_dicts **=** get_dicts("train")

**for** d **in** random**.**sample(dataset_dicts, 10):

img **=** cv2**.**imread(d["file_name"])

*# print(json.dumps(d, indent=2))*

visualizer **=** Visualizer(img[:, :, ::**-**1], scale**=**0.5)

**try**:

out **=** visualizer**.**draw_dataset_dict(d)

plot(out**.**get_image()[:, :, ::**-**1])

**except** Exception:

plot(img)


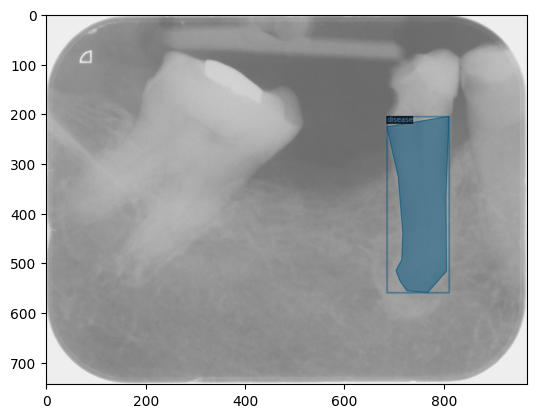

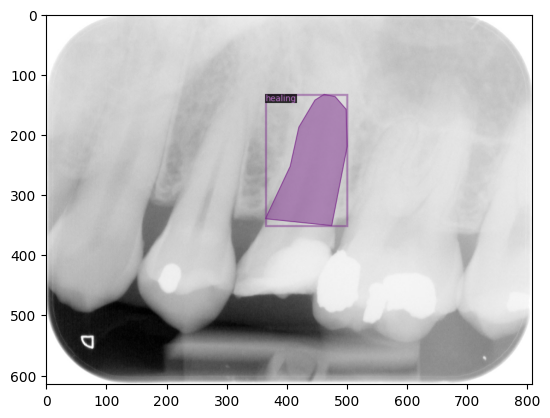

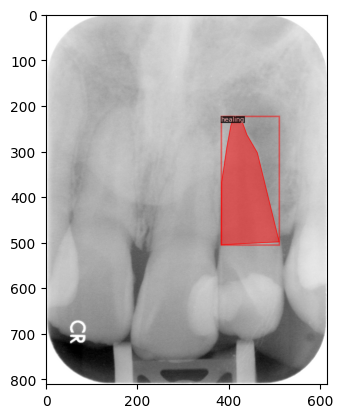

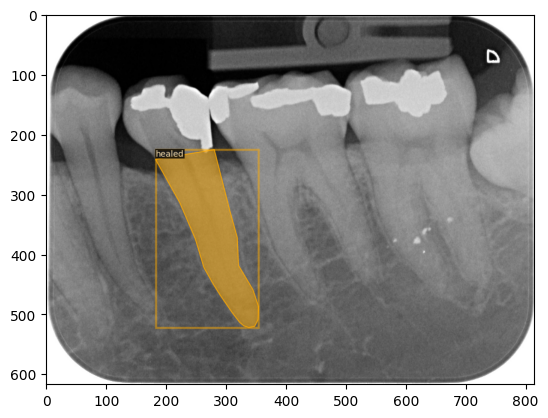
In [38]:

output_path **=** os**.**path**.**join("output", "maskRcnn")

os**.**makedirs(output_path, exist_ok**=True**)

In [39]:

**from** detectron2.engine **import** DefaultTrainer, launch

cfg **=** get_cfg()

cfg**.**merge_from_file(model_zoo**.**get_config_file("COCO-InstanceSegmentation/mask_rcnn_R_50_C4_1x.yaml"))

cfg**.**DATASETS**.**TRAIN **=** ("oral_{}_train"**.**format(version),)

cfg**.**DATASETS**.**TEST **=** ("oral_{}_validate"**.**format(version),)

cfg**.**DATALOADER**.**NUM_WORKERS **=** 2

cfg**.**NUM_GPUS **=** 1

cfg**.**MODEL**.**WEIGHTS **=** model_zoo**.**get_checkpoint_url("COCO-InstanceSegmentation/mask_rcnn_R_50_C4_1x.yaml")

cfg**.**MODEL**.**ROI_HEADS**.**NUM_CLASSES **=** len(meta**.**get('class'))

os**.**makedirs(output_path, exist_ok**=True**)

cfg**.**OUTPUT_DIR **=** output_path

*# skip blank annotation*

cfg**.**DATALOADER**.**FILTER_EMPTY_ANNOTATIONS **=** **False**

In [40]:

cfg**.**SOLVER**.**IMS_PER_BATCH **=** 4

cfg**.**SOLVER**.**BASE_LR **=** 0.00025

cfg**.**SOLVER**.**MAX_ITER **=** 20000

cfg**.**MODEL**.**ROI_HEADS**.**BATCH_SIZE_PER_IMAGE **=** 64

In [41]:

trainer **=** DefaultTrainer(cfg)

trainer**.**resume_or_load(resume**=True**)

trainer**.**train()

[07/25 11:43:19 d2.engine.defaults]: Model:

GeneralizedRCNN(

(backbone): ResNet(

(stem): BasicStem(

(conv1): Conv2d(

3, 64, kernel_size=(7, 7), stride=(2, 2), padding=(3, 3), bias=False

(norm): FrozenBatchNorm2d(num_features=64, eps=1e-05)

)

)

(res2): Sequential(

(0): BottleneckBlock(

(shortcut): Conv2d(

64, 256, kernel_size=(1, 1), stride=(1, 1), bias=False

(norm): FrozenBatchNorm2d(num_features=256, eps=1e-05)

)

(conv1): Conv2d(

64, 64, kernel_size=(1, 1), stride=(1, 1), bias=False

(norm): FrozenBatchNorm2d(num_features=64, eps=1e-05)

)

(conv2): Conv2d(

64, 64, kernel_size=(3, 3), stride=(1, 1), padding=(1, 1), bias=False

(norm): FrozenBatchNorm2d(num_features=64, eps=1e-05)

)

(conv3): Conv2d(

64, 256, kernel_size=(1, 1), stride=(1, 1), bias=False

(norm): FrozenBatchNorm2d(num_features=256, eps=1e-05)

)

)

(1): BottleneckBlock(

(conv1): Conv2d(

256, 64, kernel_size=(1, 1), stride=(1, 1), bias=False

(norm): FrozenBatchNorm2d(num_features=64, eps=1e-05)

)

(conv2): Conv2d(

64, 64, kernel_size=(3, 3), stride=(1, 1), padding=(1, 1), bias=False

(norm): FrozenBatchNorm2d(num_features=64, eps=1e-05)

)

(conv3): Conv2d(

64, 256, kernel_size=(1, 1), stride=(1, 1), bias=False

(norm): FrozenBatchNorm2d(num_features=256, eps=1e-05)

)

)

(2): BottleneckBlock(

(conv1): Conv2d(

256, 64, kernel_size=(1, 1), stride=(1, 1), bias=False

(norm): FrozenBatchNorm2d(num_features=64, eps=1e-05)

)

(conv2): Conv2d(

64, 64, kernel_size=(3, 3), stride=(1, 1), padding=(1, 1), bias=False

(norm): FrozenBatchNorm2d(num_features=64, eps=1e-05)

)

(conv3): Conv2d(

64, 256, kernel_size=(1, 1), stride=(1, 1), bias=False

(norm): FrozenBatchNorm2d(num_features=256, eps=1e-05)

)

)

)

(res3): Sequential(

(0): BottleneckBlock(

(shortcut): Conv2d(

256, 512, kernel_size=(1, 1), stride=(2, 2), bias=False

(norm): FrozenBatchNorm2d(num_features=512, eps=1e-05)

)

(conv1): Conv2d(

256, 128, kernel_size=(1, 1), stride=(2, 2), bias=False

(norm): FrozenBatchNorm2d(num_features=128, eps=1e-05)

)

(conv2): Conv2d(

128, 128, kernel_size=(3, 3), stride=(1, 1), padding=(1, 1), bias=False

(norm): FrozenBatchNorm2d(num_features=128, eps=1e-05)

)

(conv3): Conv2d(

128, 512, kernel_size=(1, 1), stride=(1, 1), bias=False

(norm): FrozenBatchNorm2d(num_features=512, eps=1e-05)

)

)

(1): BottleneckBlock(

(conv1): Conv2d(

512, 128, kernel_size=(1, 1), stride=(1, 1), bias=False

(norm): FrozenBatchNorm2d(num_features=128, eps=1e-05)

)

(conv2): Conv2d(

128, 128, kernel_size=(3, 3), stride=(1, 1), padding=(1, 1), bias=False

(norm): FrozenBatchNorm2d(num_features=128, eps=1e-05)

)

(conv3): Conv2d(

128, 512, kernel_size=(1, 1), stride=(1, 1), bias=False

(norm): FrozenBatchNorm2d(num_features=512, eps=1e-05)

)

)

(2): BottleneckBlock(

(conv1): Conv2d(

512, 128, kernel_size=(1, 1), stride=(1, 1), bias=False

(norm): FrozenBatchNorm2d(num_features=128, eps=1e-05)

)

(conv2): Conv2d(

128, 128, kernel_size=(3, 3), stride=(1, 1), padding=(1, 1), bias=False

(norm): FrozenBatchNorm2d(num_features=128, eps=1e-05)

)

(conv3): Conv2d(

128, 512, kernel_size=(1, 1), stride=(1, 1), bias=False

(norm): FrozenBatchNorm2d(num_features=512, eps=1e-05)

)

)

(3): BottleneckBlock(

(conv1): Conv2d(

512, 128, kernel_size=(1, 1), stride=(1, 1), bias=False

(norm): FrozenBatchNorm2d(num_features=128, eps=1e-05)

)

(conv2): Conv2d(

128, 128, kernel_size=(3, 3), stride=(1, 1), padding=(1, 1), bias=False

(norm): FrozenBatchNorm2d(num_features=128, eps=1e-05)

)

(conv3): Conv2d(

128, 512, kernel_size=(1, 1), stride=(1, 1), bias=False

(norm): FrozenBatchNorm2d(num_features=512, eps=1e-05)

)

)

)

(res4): Sequential(

(0): BottleneckBlock(

(shortcut): Conv2d(

512, 1024, kernel_size=(1, 1), stride=(2, 2), bias=False

(norm): FrozenBatchNorm2d(num_features=1024, eps=1e-05)

)

(conv1): Conv2d(

512, 256, kernel_size=(1, 1), stride=(2, 2), bias=False

(norm): FrozenBatchNorm2d(num_features=256, eps=1e-05)

)

(conv2): Conv2d(

256, 256, kernel_size=(3, 3), stride=(1, 1), padding=(1, 1), bias=False

(norm): FrozenBatchNorm2d(num_features=256, eps=1e-05)

)

(conv3): Conv2d(

256, 1024, kernel_size=(1, 1), stride=(1, 1), bias=False

(norm): FrozenBatchNorm2d(num_features=1024, eps=1e-05)

)

)

(1): BottleneckBlock(

(conv1): Conv2d(

1024, 256, kernel_size=(1, 1), stride=(1, 1), bias=False

(norm): FrozenBatchNorm2d(num_features=256, eps=1e-05)

)

(conv2): Conv2d(

256, 256, kernel_size=(3, 3), stride=(1, 1), padding=(1, 1), bias=False

(norm): FrozenBatchNorm2d(num_features=256, eps=1e-05)

)

(conv3): Conv2d(

256, 1024, kernel_size=(1, 1), stride=(1, 1), bias=False

(norm): FrozenBatchNorm2d(num_features=1024, eps=1e-05)

)

)

(2): BottleneckBlock(

(conv1): Conv2d(

1024, 256, kernel_size=(1, 1), stride=(1, 1), bias=False

(norm): FrozenBatchNorm2d(num_features=256, eps=1e-05)

)

(conv2): Conv2d(

256, 256, kernel_size=(3, 3), stride=(1, 1), padding=(1, 1), bias=False

(norm): FrozenBatchNorm2d(num_features=256, eps=1e-05)

)

(conv3): Conv2d(

256, 1024, kernel_size=(1, 1), stride=(1, 1), bias=False

(norm): FrozenBatchNorm2d(num_features=1024, eps=1e-05)

)

)

(3): BottleneckBlock(

(conv1): Conv2d(

1024, 256, kernel_size=(1, 1), stride=(1, 1), bias=False

(norm): FrozenBatchNorm2d(num_features=256, eps=1e-05)

)

(conv2): Conv2d(

256, 256, kernel_size=(3, 3), stride=(1, 1), padding=(1, 1), bias=False

(norm): FrozenBatchNorm2d(num_features=256, eps=1e-05)

)

(conv3): Conv2d(

256, 1024, kernel_size=(1, 1), stride=(1, 1), bias=False

(norm): FrozenBatchNorm2d(num_features=1024, eps=1e-05)

)

)

(4): BottleneckBlock(

(conv1): Conv2d(

1024, 256, kernel_size=(1, 1), stride=(1, 1), bias=False

(norm): FrozenBatchNorm2d(num_features=256, eps=1e-05)

)

(conv2): Conv2d(

256, 256, kernel_size=(3, 3), stride=(1, 1), padding=(1, 1), bias=False

(norm): FrozenBatchNorm2d(num_features=256, eps=1e-05)

)

(conv3): Conv2d(

256, 1024, kernel_size=(1, 1), stride=(1, 1), bias=False

(norm): FrozenBatchNorm2d(num_features=1024, eps=1e-05)

)

)

(5): BottleneckBlock(

(conv1): Conv2d(

1024, 256, kernel_size=(1, 1), stride=(1, 1), bias=False

(norm): FrozenBatchNorm2d(num_features=256, eps=1e-05)

)

(conv2): Conv2d(

256, 256, kernel_size=(3, 3), stride=(1, 1), padding=(1, 1), bias=False

(norm): FrozenBatchNorm2d(num_features=256, eps=1e-05)

)

(conv3): Conv2d(

256, 1024, kernel_size=(1, 1), stride=(1, 1), bias=False

(norm): FrozenBatchNorm2d(num_features=1024, eps=1e-05)

)

)

)

)

(proposal_generator): RPN(

(rpn_head): StandardRPNHead(

(conv): Conv2d(

1024, 1024, kernel_size=(3, 3), stride=(1, 1), padding=(1, 1)

(activation): ReLU()

)

(objectness_logits): Conv2d(1024, 15, kernel_size=(1, 1), stride=(1, 1))

(anchor_deltas): Conv2d(1024, 60, kernel_size=(1, 1), stride=(1, 1))

)

(anchor_generator): DefaultAnchorGenerator(

(cell_anchors): BufferList()

)

)

(roi_heads): Res5ROIHeads(

(pooler): ROIPooler(

(level_poolers): ModuleList(

(0): ROIAlign(output_size=(14, 14), spatial_scale=0.0625, sampling_ratio=0, aligned=True)

)

)

(res5): Sequential(

(0): BottleneckBlock(

(shortcut): Conv2d(

1024, 2048, kernel_size=(1, 1), stride=(2, 2), bias=False

(norm): FrozenBatchNorm2d(num_features=2048, eps=1e-05)

)

(conv1): Conv2d(

1024, 512, kernel_size=(1, 1), stride=(2, 2), bias=False

(norm): FrozenBatchNorm2d(num_features=512, eps=1e-05)

)

(conv2): Conv2d(

512, 512, kernel_size=(3, 3), stride=(1, 1), padding=(1, 1), bias=False

(norm): FrozenBatchNorm2d(num_features=512, eps=1e-05)

)

(conv3): Conv2d(

512, 2048, kernel_size=(1, 1), stride=(1, 1), bias=False

(norm): FrozenBatchNorm2d(num_features=2048, eps=1e-05)

)

)

(1): BottleneckBlock(

(conv1): Conv2d(

2048, 512, kernel_size=(1, 1), stride=(1, 1), bias=False

(norm): FrozenBatchNorm2d(num_features=512, eps=1e-05)

)

(conv2): Conv2d(

512, 512, kernel_size=(3, 3), stride=(1, 1), padding=(1, 1), bias=False

(norm): FrozenBatchNorm2d(num_features=512, eps=1e-05)

)

(conv3): Conv2d(

512, 2048, kernel_size=(1, 1), stride=(1, 1), bias=False

(norm): FrozenBatchNorm2d(num_features=2048, eps=1e-05)

)

)

(2): BottleneckBlock(

(conv1): Conv2d(

2048, 512, kernel_size=(1, 1), stride=(1, 1), bias=False

(norm): FrozenBatchNorm2d(num_features=512, eps=1e-05)

)

(conv2): Conv2d(

512, 512, kernel_size=(3, 3), stride=(1, 1), padding=(1, 1), bias=False

(norm): FrozenBatchNorm2d(num_features=512, eps=1e-05)

)

(conv3): Conv2d(

512, 2048, kernel_size=(1, 1), stride=(1, 1), bias=False

(norm): FrozenBatchNorm2d(num_features=2048, eps=1e-05)

)

)

)

(box_predictor): FastRCNNOutputLayers(

(cls_score): Linear(in_features=2048, out_features=4, bias=True)

(bbox_pred): Linear(in_features=2048, out_features=12, bias=True)

)

(mask_head): MaskRCNNConvUpsampleHead(

(deconv): ConvTranspose2d(2048, 256, kernel_size=(2, 2), stride=(2, 2))

(deconv_relu): ReLU()

(predictor): Conv2d(256, 3, kernel_size=(1, 1), stride=(1, 1))

)

)

)

In [42]:

cfg**.**MODEL**.**WEIGHTS **=** os**.**path**.**join(cfg**.**OUTPUT_DIR, "model_final.pth") *# path to the model we just trained*

cfg**.**MODEL**.**ROI_HEADS**.**SCORE_THRESH_TEST **=** 0.75

predictor **=** DefaultPredictor(cfg)

In [43]:

**with** open(os**.**path**.**join(output_path,'metrics.json'), 'r') **as** f:

metrics **=** json**.**loads("["**+** (f**.**read()**.**replace("}","},")) **+** "{}]")

In [44]:

train_loss **=** []

**for** d **in** metrics:

**if** 'total_loss' **in** d **and** 'iteration' **in** d:

train_loss**.**append({

'iteration' : d['iteration'],

'train_loss': d['total_loss']

})

train_loss_df **=** pd**.**DataFrame(train_loss)

train_loss_df

Out[44]:

|  | **iteration** | **train_loss** |
| --- | --- | --- |
| **0** | 19 | 1.813716 |
| **1** | 39 | 1.646061 |
| **2** | 59 | 1.445516 |
| **3** | 79 | 1.145758 |
| **4** | 99 | 0.972889 |
| **...** | ... | ... |
| **1013** | 19919 | 0.294158 |
| **1014** | 19939 | 0.259275 |
| **1015** | 19959 | 0.287467 |
| **1016** | 19979 | 0.289028 |
| **1017** | 19999 | 0.304558 |


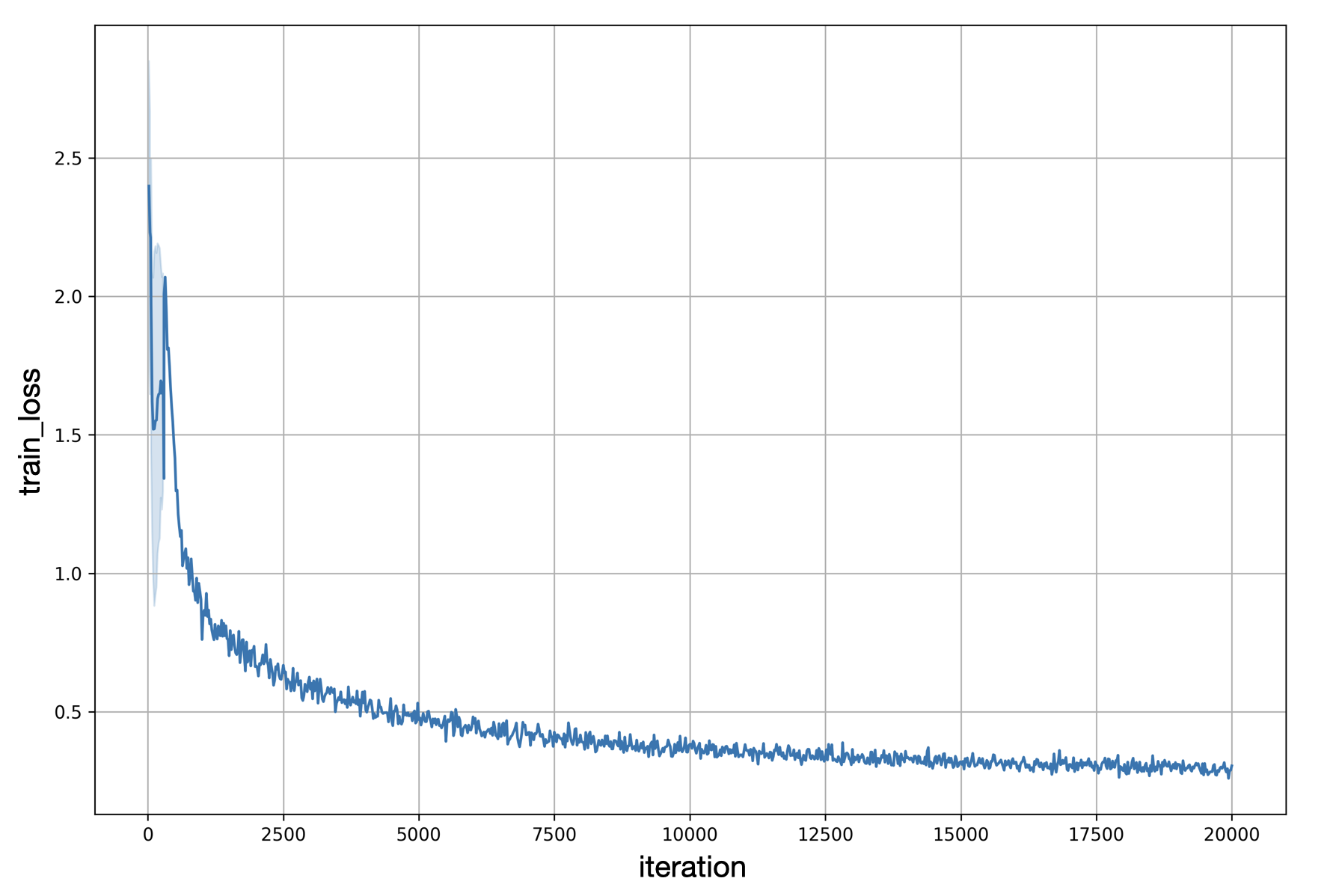


In [45]:

model_name **=** "mask_rcnn"

plt**.**figure(figsize**=**(12,8))

plt**.**grid()

plt**.**title('train_loss_{}'**.**format(model_name))

fig **=** sns**.**lineplot(x**=**'iteration', y**=**'train_loss', data**=**train_loss_df)

fig**.**figure**.**savefig(os**.**path**.**join(output_path, "train_loss_{}.png"**.**format(model_name)), dpi**=**300)

In [46]:

**try**:

MetadataCatalog**.**remove('oral_{}_test'**.**format(version))

DatasetCatalog**.**remove('oral_{}_test'**.**format(version))

**except** Exception:

**pass**

In [47]:

**for** d **in** ["test"]:

DatasetCatalog**.**register("oral_{}_{}"**.**format(version, d), **lambda** d**=**d: get_dicts(d))

MetadataCatalog**.**get("oral_{}_{}"**.**format(version, d))**.**set(thing_classes**=**meta**.**get('class',[]))

metadata_test **=** MetadataCatalog**.**get("oral_{}_test"**.**format(version))

In [48]:

**from** detectron2.utils.visualizer **import** ColorMode

*# dataset_dicts = get_dicts("test")*

dataset_dicts **=** get_dicts("test")

**for** d **in** random**.**sample(dataset_dicts, 10):

im **=** cv2**.**imread(d["file_name"])

outputs **=** predictor(im) *# format is documented at https://detectron2.readthedocs.io/tutorials/models.html#model-output-format*

v **=** Visualizer(im[:, :, ::**-**1],

metadata**=**metadata_test,

scale**=**0.5,

instance_mode**=**ColorMode**.**IMAGE_BW *# remove the colors of unsegmented pixels. This option is only available for segmentation models*

)

pred_out **=** v**.**draw_instance_predictions(outputs["instances"]**.**to("cpu"))

visualizer **=** Visualizer(im[:, :, ::**-**1], metadata**=**metadata, scale**=**0.5)

**try**:

gt_out **=** visualizer**.**draw_dataset_dict(d)

**except** Exception:

gt_out **=** **None**

fig, ax **=** plt**.**subplots(1,2, figsize**=**(12,12))

**if** gt_out **is** **not** **None**:

ax[0]**.**imshow(cv2plt(gt_out**.**get_image()[:, :, ::**-**1]))

**else**:

ax[0]**.**imshow(cv2plt(im))

ax[0]**.**set_title('groundtruths')

ax[1]**.**imshow(cv2plt(pred_out**.**get_image()[:, :, ::**-**1]))

ax[1]**.**set_title('detection')

plt**.**show()

Out [48]:


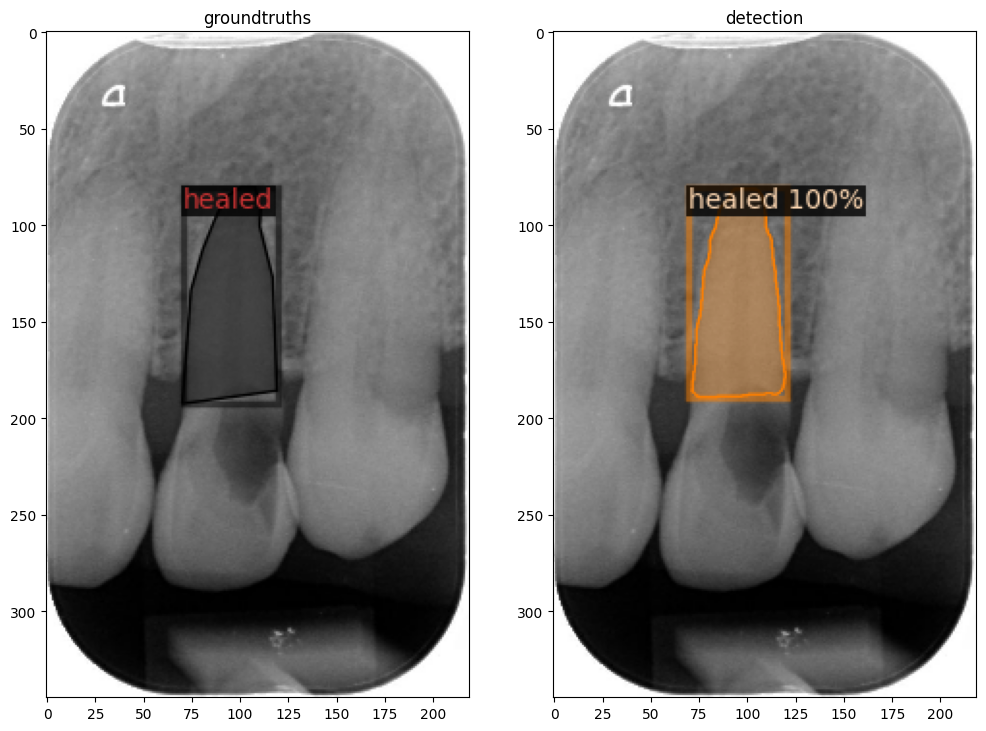

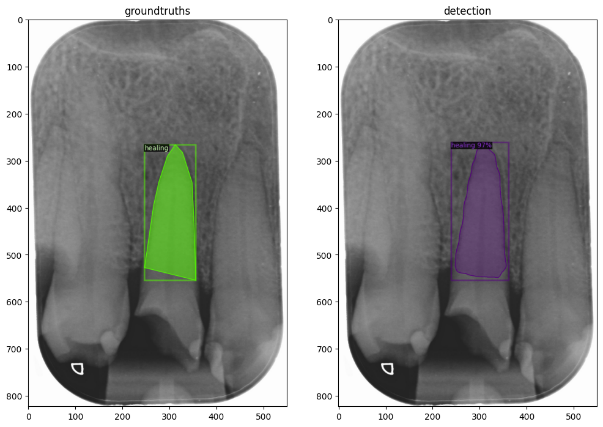


|  | **disease** | **healed** | **healing** |
| --- | --- | --- | --- |
| **Precision** | 0.921609 | 0.884872 | 0.841487 |
| **Recall** | 0.881429 | 0.924444 | 0.862143 |
| **F1** | 0.903242 | 0.901057 | 0.821216 |

In [49]:

*# pip install openpyxl*

In [50]:

**for** cls **in** extract_result:

plt**.**figure(figsize**=**(12,8))

plt**.**grid()

plt**.**title("Precision x Recall Curve\n \nClass: {} AP: {:.2f}\nAUC: {:.2f}"**.**format(cls,extract_result[cls]['Precision'], extract_result[cls]['auc']))

plt**.**plot(results[cls]['recall'], results[cls]['precision'], label**=**'precision')

plt**.**plot(results[cls]['interpolated_recall'], results[cls]['interpolated_precision'], "--", label**=**'interpolated precision')

plt**.**xlabel("recall")

plt**.**ylabel("precision")

plt**.**legend()

plt**.**savefig(os**.**path**.**join("output","maskRcnn","Precision_Recall_Curve_{}.png"**.**format(cls)))

plt**.**show()

Out [50]:
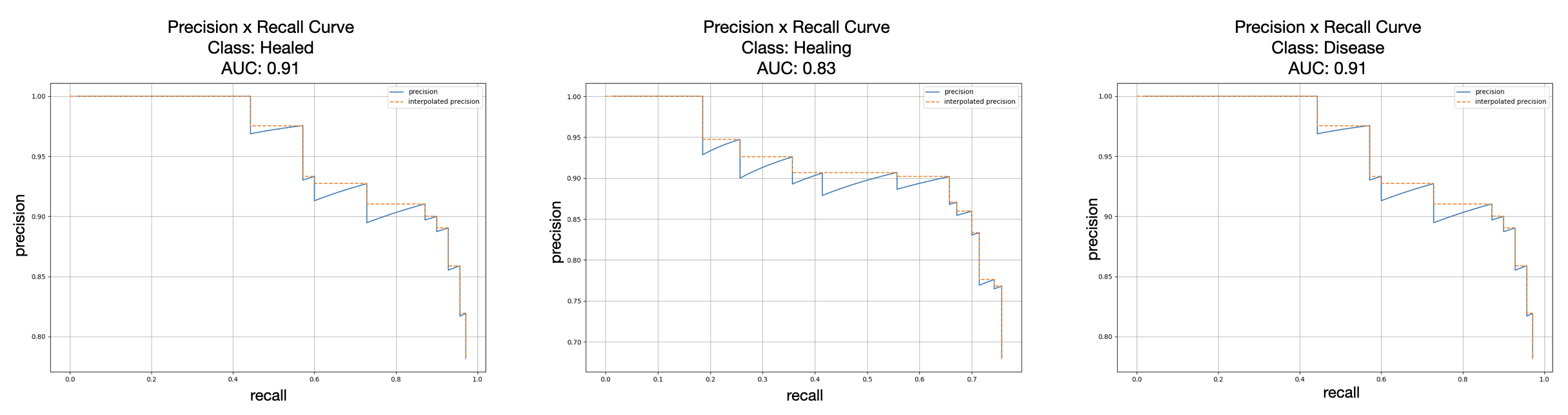

Supplement: S1 File — (DOCX) [file pone.0310925.s001.docx]
